# Subtype distribution and long-term titer fluctuation patterns of serum anti-Epstein–Barr virus antibodies in a non-nasopharyngeal carcinoma population from an endemic area in South China: a cohort study

**DOI:** 10.1186/s40880-016-0130-2

**Published:** 2016-08-15

**Authors:** Jin-Lin Du, Sui-Hong Chen, Qi-Hong Huang, Shang-Hang Xie, Yan-Fang Ye, Rui Gao, Jie Guo, Meng-Jie Yang, Qing Liu, Ming-Huang Hong, Su-Mei Cao

**Affiliations:** 1 State Key Laboratory of Oncology in South China, Collaborative Innovation Center for Cancer Medicine, Sun Yat-sen University Cancer Center, Guangzhou, 510060 Guangdong P. R. China; 2 Department of Cancer Prevention Research, Sun Yat-sen University Cancer Center, Guangzhou, 510060 Guangdong P. R. China; 3 School of Public Health, Sun Yat-sen University, Guangzhou, 510080 Guangdong P. R. China; 4 School of Public Health, Guangdong Medical University, Dongguan, 523808 Guangdong P. R. China; 5 Sihui Cancer Institute, Sihui, 526200 Guangdong P. R. China; 6 Department of Clinical Trial Center, Sun Yat-sen University Cancer Center, Guangzhou, 510060 Guangdong P. R. China

**Keywords:** Epstein–Barr virus, Nasopharyngeal carcinoma, Cohort study, Mass screening, Fluctuation

## Abstract

**Background:**

Serum immunoglobulin A antibodies against Epstein–Barr virus (EBV), viral capsid antigen (VCA-IgA) and early antigen (EA-IgA), are used to screen for nasopharyngeal carcinoma (NPC) in endemic areas. However, their routine use has been questioned because of a lack of specificity. This study aimed to determine the distributions of different subtypes of antibody and to illustrate how the natural variation patterns affect the specificity of screening in non-NPC participants.

**Methods:**

The distribution of baseline VCA-IgA was analyzed between sexes and across 10-year age groups in 18,286 non-NPC participants using Chi square tests. Fluctuations in the VCA-IgA level were assessed in 1056 non-NPC participants with at least two retests in the first 5-year period (1987–1992) after the initial screening using the Kaplan–Meier method.

**Results:**

The titers of VCA-IgA increased with age (*P* < 0.001). Using a previous serological definition of high NPC risk, nasopharyngeal endoscopy and/or nasopharyngeal biopsy would be recommended in 55.5% of the non-NPC participants with an initial VCA-IgA-positive status and in 20.6% with an initial negative status during the 5-year follow-up. However, seroconversions were common; 85.2% of the participants with a VCA-IgA-positive status at baseline converted to negative, and all VCA-IgA-negative participants changed to positive at least once during the 5-year follow-up. The EA-IgA status had a high seroconversion probability (100%) from positive to negative; however, it had a low probability (19.6%) from negative to positive.

**Conclusions:**

Age- and sex-specific cutoff titer values for serum anti-EBV antibodies as well as their specific titer fluctuation patterns should be considered when defining high NPC risk criteria for follow-up diagnostics and monitoring.

## Background

Nasopharyngeal carcinoma (NPC) is rare in most regions of the world, with an incidence of less than 1 per 100,000 person-years [1]. However, in some regions of South China and Southeast Asia, the incidence of NPC approaches 20–50 per 100,000 person-years [2, 3]. The pathogenesis is not fully understood; however, accumulated evidence indicates that genetic susceptibility, Epstein–Barr virus (EBV) infection [4], and environmental factors, such as salted-fish consumption and cigarette smoking, increase the risk of NPC development [5, 6]. NPC easily spreads to the lymph nodes and distant organs, and most patients are diagnosed only when the tumor has reached an advanced stage (stages III and IV) [7].

More than 95% of adults in some NPC endemic areas are infected with EBV. Infection typically occurs in childhood, and the virus remains in a latent phase within resting memory B cells under the strict monitoring of the immune system [8, 9]. However, the virus may be periodically reactivated in response to environmental stress [5]. Several studies have reported that smoking [5], Cantonese-style salted fish [10], and Chinese herbs [11], all of which contain EBV-inducing substances, may drive EBV into the lytic cycle [12].

Given the unclear etiology of NPC, primary prevention remains difficult, and the main strategy for reducing NPC mortality is to identify the disease at an early stage [13]. In the preclinical phase, serum antibodies against EBV-related antigens remain increased for an average of 38 months [14–16], and the serological tests for these markers are simple and inexpensive [15]. Therefore, these antibodies have been used as screening markers for NPC in endemic areas since the 1970s. The immunoglobulin A (IgA) antibodies against viral capsid antigen (VCA-IgA) and early antigen (EA-IgA) are the most popular screening markers. VCA is the structural protein expressed by EBV during the late stage of the lytic cycle following viral DNA synthesis, whereas EA is a nonstructural protein expressed during the switch from the latent phase to lytic replication before viral DNA synthesis. The two markers have distinct features for NPC screening: VCA-IgA has a higher sensitivity than EA-IgA (79.6% vs. 31.6%); however, its specificity is lower than EA-IgA (92.0% vs. 99.7%) [13]. Thus, in practice, these two markers are typically used in combination for NPC screening [15, 17].

Several prospective screening studies have demonstrated early detection rates for NPC of greater than 70% [15, 18, 19]. Nevertheless, EBV infection is ubiquitous; thus, routine use as a screening tool may have relatively low specificity, which may lead to potentially costly and invasive follow-up diagnostics [19–21]. Therefore, an investigation of the natural variations of EBV antibodies in non-NPC participants may identify particular serostatus patterns for improved NPC screening.

In this study, we used EBV VCA-IgA and EA-IgA data from a prospective, population-based screening study with no clinical evidence of NPC during a median 15.9-year follow-up in South China [17] to determine the distribution of baseline EBV VCA-IgA between sexes and among age groups as well as the long-term titer fluctuations of the VCA-IgA and EA-IgA serostatus in non-NPC participants. The aim was to identify additional factors useful for EBV antibody-based NPC screening strategies in endemic areas.

## Methods

### Study population

**Fig. 1:**
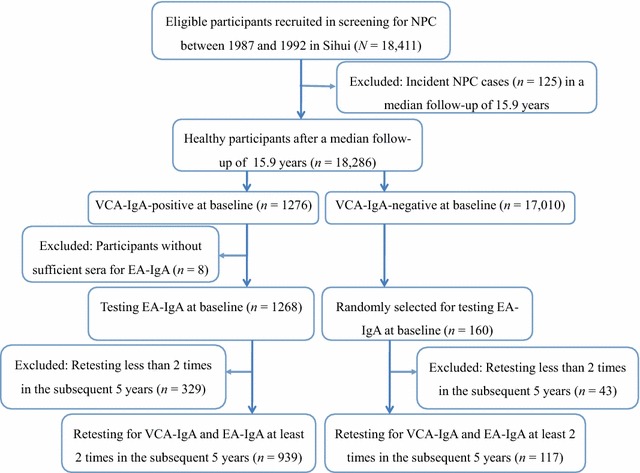
Recruitment diagram of the non-nasopharyngeal carcinoma (NPC) population for the analysis of the long-term variation trends of serum Epstein–Barr virus (EBV) antibodies. *VCA-IgA* immunoglobulin A (IgA) antibody against EBV viral capsid antigen; *EA-IgA* IgA antibody against EBV early antigen

Serological EBV VCA-IgA and EA-IgA test results and follow-up information were obtained from an NPC screening cohort from Sihui County, Guangdong Province, China [17]. Briefly, 18,411 eligible, non-NPC residents aged 30–59 years in four towns of Sihui County were recruited in 1987 and 1992 (7774 in 1987 and 10,637 in 1992) [17]. Blood samples were collected at recruitment and tested for VCA-IgA using immunoenzymatic assay [22]. Participants positive at baseline for VCA-IgA were further tested for EA-IgA. High-risk individuals, including those with a VCA-IgA titer ≥1:40 or seropositive for both markers (cutoff = 1:5), were referred for nasopharyngeal endoscopic examination and/or pathological biopsy. We followed the participants every year from the initial screening until the diagnosis of NPC, migration, death, or the end of 2007, whichever event occurred first, using record links to the Cancer Register, Cause of Death Register, the Rosters of Village Committee, and the local Public Security Bureau in Sihui. The completeness of the Sihui Cancer Register has been described in detail previously [17]. A total of 125 participants with NPC were identified at screening, leaving a total of 18,286 non-NPC participants in the present study. During a median 15.9 years of follow-up, 1276 non-NPC participants with a baseline VCA-IgA-positive status were advised to be repeatedly tested for VCA-IgA and EA-IgA every year. A total of 939 participants VCA-IgA-positive at baseline (73.6%) were retested at least twice during the first 5-year follow-up. In addition, 160 of 17,010 participants VCA-IgA-negative at baseline were randomly selected to be retested for both screening markers every year, and 117 of these participants (73.1%) were retested at least twice in the first 5 years. Finally, 1056 non-NPC participants with at least two VCA-IgA and EA-IgA retests in the initial 5 years were included in the final analysis (Fig. 1).

Permission to use the data from the screening program and follow-up results was granted by the Institutional Ethics Review Board of Sun Yat-sen University Cancer Center (SYSUCC) (No. YP2009169).

### Serological analysis

At baseline and each subsequent follow-up year, eligible participants were invited to donate 3 mL of blood to determine the VCA-IgA and EA-IgA statuses. Serological tests using immunoenzymatic assays were performed in the laboratory of the SYSUCC as described previously [22]. A titer of 1:5 was defined as positive for VCA-IgA and EA-IgA. Titers were further classified into subgroups according to the maximum dilution of serum [17], with a VCA-IgA titer ≥1:40 or both VCA-IgA- and EA-IgA-positive (cutoff = 1:5) defined as high risk for NPC.

Quality control with a pooled serum sample as the standard has been used in every test conducted by the SYSUCC since the 1980s. The coefficient of variation (CV) of the assay for VCA-IgA over 8 years (1993–2000) was 8.37% [17].

### Statistical analysis

The different serum levels of VCA-IgA were compared among 10-year age groups, between sexes, and according to the recruitment time using Chi square tests. Linear trend tests for the association between age and VCA-IgA were performed with bidirectionally ordered variables.

The seroconversion of VCA-IgA was defined as a non-NPC participant with a VCA-IgA-negative status (cutoff = 1:5) at baseline changed to positive or with a VCA-IgA-positive status at baseline changed to negative at least once in the subsequent 5-year follow-up. The seroconversion of EA-IgA was defined the same as that of VCA-IgA. In the cumulative probability analysis, only the first change in status (from baseline negative to positive or from baseline positive to negative) was considered. The cumulative probability of seroconversion and the median duration of the original serum status were derived via the Kaplan–Meier method, with log-rank tests used to identify differences between sexes and the serum EBV status groups for the specific screening marker. Person-time was calculated from the baseline test to the first serum EBV change. The participants whose serum EBV status did not convert by the final visit at 5 years after screening were censored in the Kaplan–Meier analysis.

In the cumulative probability analysis of the participants who met the high-risk criteria, the outcome event was defined as a non-NPC participant with a baseline VCA-IgA ≥1:40 or both VCA-IgA and EA-IgA ≥1:5 at least once in the subsequent 5-year follow-up. The time to meet the high-risk criteria was calculated from the baseline test to the first visit at which a high risk criterion was identified. The cumulative probability was calculated using the Kaplan–Meier method.

All statistical analyses, unless otherwise noted, were performed using IBM Statistical Package for the Social Sciences Statistics 20 (IBM Corp, Chicago, IL, USA). All statistical tests were two-sided, and *P* < 0.05 was considered significant.

## Results

### Distribution of EBV antibodies in non-NPC participants

A total of 18,286 non-NPC participants in the screening cohort were included for baseline EBV antibody analysis. The baseline EBV VCA-IgA seropositive rate was 7.0% (1276/18,286). As a result of insufficient sera for 8 participants, only 1268 participants were tested for EA-IgA, and 3.2% (41/1268) of them were also positive for EA-IgA. The baseline seropositive rate of VCA-IgA was higher in males than in females (7.6% vs. 6.6%), and the difference was significant after adjusting for age (Mantel–Haenszel stratified χ^2^ = 7.286, *P* = 0.007). The VCA-IgA level increased significantly with age (*P*_trends_ < 0.05) in both males (χ^2^ = 11.844) and females (χ^2^ = 15.475) (Table 1). Using 1:40 as the cutoff value for a high NPC risk, the VCA-IgA positive rate in the 30–39 and 50–59 year age groups increased from 0.3% to 0.6% in males and from 0.4% to 0.6% in females.

**Table 1:** Baseline VCA-IgA distribution in 18,286 non-nasopharyngeal carcinoma (NPC) participants by age and sex

Sex	Age (years)	No. of participants	VCA-IgA [cases (%)]					χ^2^	*P*
			–	1:5	1:10	1:20	≥1:40		
Male	30–39	3063	2861 (93.4)	108 (3.5)	69 (2.3)	16 (0.5)	9 (0.3)		
	40–49	2196	2022 (92.1)	68 (3.1)	76 (3.5)	23 (1.0)	7 (0.3)	11.844	0.001
	50–59	1740	1584 (91.0)	73 (4.2)	57 (3.3)	16 (0.9)	10 (0.6)		
Female	30–39	5541	5225 (94.3)	139 (2.5)	129 (2.3)	27 (0.5)	21 (0.4)		
	40–49	3070	2855 (93.0)	93 (3.0)	83 (2.7)	24 (0.8)	15 (0.5)	15.475	0.000
	50–59	2676	2463 (92.0)	96 (3.6)	71 (2.7)	30 (1.1)	16 (0.6)		

*VCA-IgA* immunoglobulin A (IgA) antibodies against viral capsid antigen of Epstein–Barr virus (EBV)

**Fig. 2:**
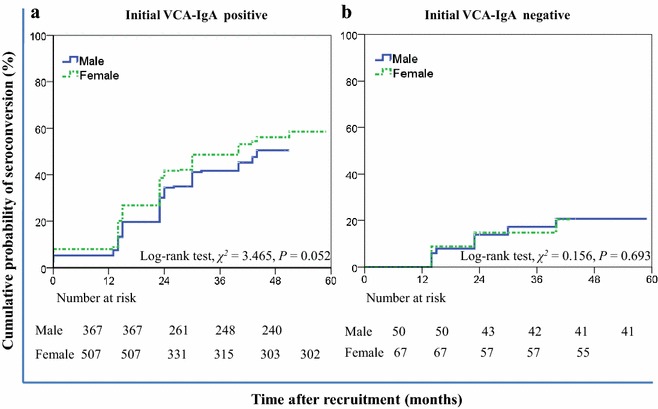
Cumulative probability for the male and female participants with an initial VCA-IgA-positive or -negative status who met the high risk criteria in the first 5-year follow-up. **a** Cumulative probabilities of seroconversion for meeting any of the high risk criteria in the participants with an initial VCA-IgA-positive status were not different between males and females; **b** cumulative probabilities of seroconversion for meeting any of the high risk criteria in the participants with an initial VCA-IgA-negative status were similar for males and females

A total of 1056 participants were tested for VCA-IgA and EA-IgA at least twice after the initial screening, with 939 VCA-IgA-positive and 117 VCA-IgA-negative participants at baseline (Table 2). There was no difference in the sex ratio or age group distribution between the baseline VCA-IgA-positive and -negative participants. Using a VCA-IgA ≥1:40 or both VCA-IgA- and EA-IgA-positive (cutoff = 1:5) as the threshold for nasopharyngeal endoscopy and/or pathological examination referral following NPC screening, the 5-year cumulative probability of seroconversion was 55.5% [95% confidence interval (CI) 49.4%–61.6%] for the participants with an initial VCA-IgA-positive status and 20.6% (95% CI 12.4%–28.8%) for the participants with an initial VCA-IgA-negative status. The 5-year cumulative probabilities for meeting either high-risk criterion were similar for males and females (Fig. 2). In the participants with an initial VCA-IgA-positive status, the cumulative probability of seroconversion was slightly higher in females than in males [56.0% (95% CI 49.7%–62.3%) vs. 50.4% (95% CI 41.6%–59.2%), *P* = 0.052]. In the participants with an initial VCA-IgA-negative status, the cumulative probabilities did not differ between males and females (20.6% (95% CI 8.1%–33.1%) vs. 20.4% (95% CI 9.6%–31.2%), *P* = 0.693].

**Table 2:** Baseline VCA-IgA-positive and -negative statuses in 1056 non-NPC participants by age and sex

Subgroup	VCA-IgA-negative	VCA-IgA-positive	χ^2^	*P*
Gender				
Male	50	388	0.086	0.770
Female	67	551		
Age (years)				
30–39	44	392	1.230	0.541
40–49	43	299		
50–59	30	248

### Titer fluctuation patterns of EBV antibodies in the non-NPC population

**Fig. 3:**
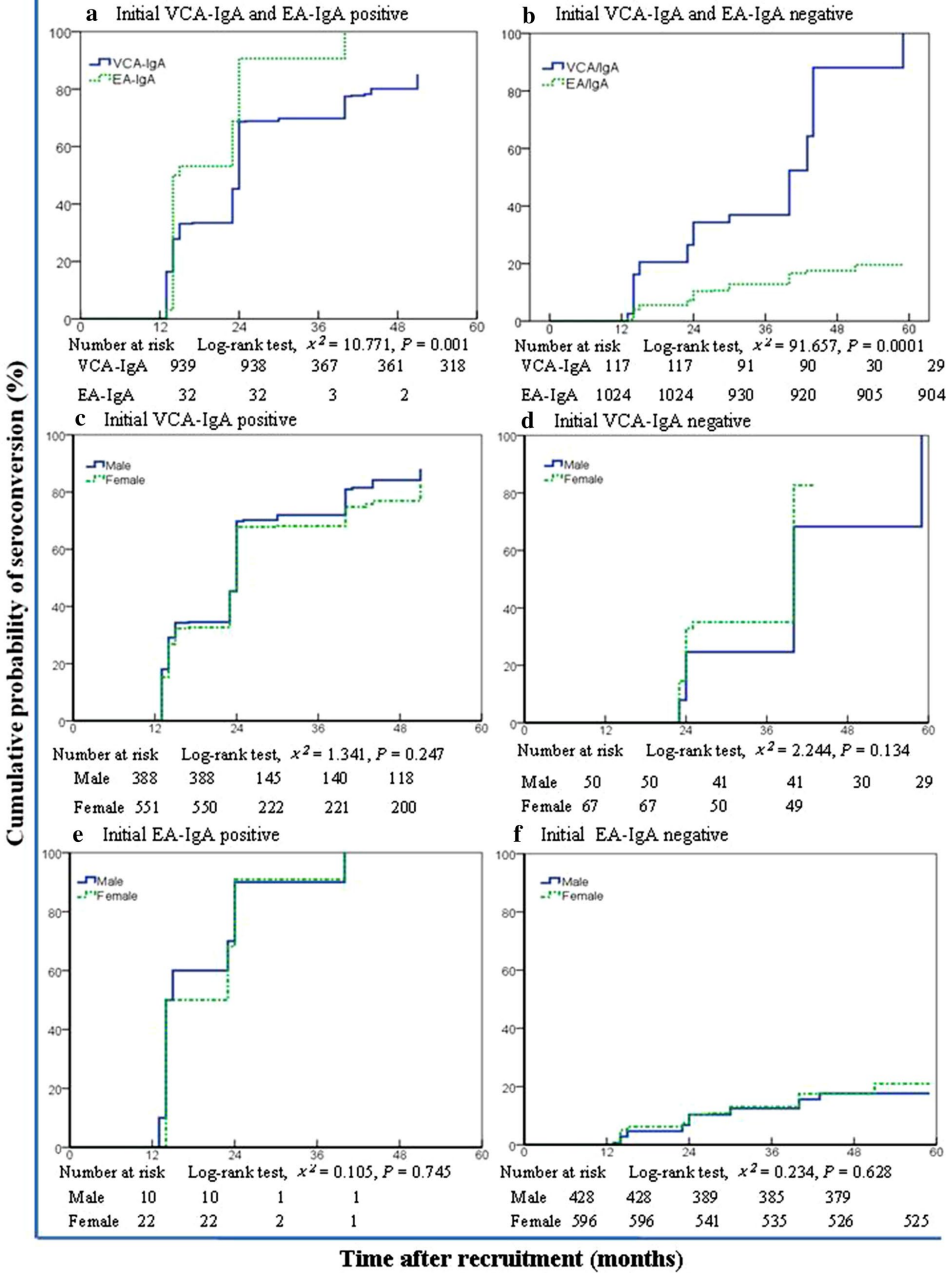
Kaplan–Meier estimates of the cumulative probability of seroconversion for the non-NPC participants with different baseline serum statuses of VCA-IgA and EA-IgA in the first 5-year follow-up. **a** Cumulative probability of seroconversion from positive to negative for VCA-IgA differed from that for EA-IgA. **b** Cumulative probability of seroconversion from negative to positive for VCA-IgA was higher than that for EA-IgA. **c** Cumulative probabilities of seroconversion from positive to negative for VCA-IgA were similar between males and females. **d** Cumulative probability of seroconversion from negative to positive for VCA-IgA in males resembled the probability in females. **e** Cumulative probabilities of seroconversion from positive to negative for EA-IgA did not differ between males and females. **f** Cumulative probabilities of seroconversion from negative to positive for EA-IgA did not differ between males and females

Long-term seroconversions were common for both VCA-IgA and EA-IgA (Fig. 3). In the 939 initial VCA-IgA-positive participants who received at least two retests, the median VCA-IgA-positive duration was 24 months (95% CI 23.3–24.7 months), and the cumulative probability of seroconversion within 5 years was 85.2% (95% CI 81.5%–88.9%) (Fig. 3a). In the 117 participants with an initial VCA-IgA-negative status retested at least twice, the median VCA-IgA-negative duration was 40 months (95% CI 35.1–44.9 months), and the 5-year cumulative probability of seroconversion was 100.0% (Fig. 3b).

In the non-NPC participants who met the high-risk criteria at baseline, the cumulative probability of seroconversion within 5 years was also high, with 33.8% (95% CI 25.4%–42.3%) for the participants with a VCA-IgA ≥1:40, 42.0% (95% CI 31.2%–52.8%) for those with both VCA-IgA- and EA-IgA-positive, and 44.5% (95% CI 36.1%–52.9%) for those fulfilling one of the two high-risk criteria that indicated conversion within 5 years.

In the 939 participants with an initial VCA-IgA-positive status and retested at least twice in the first 5-year follow-up, 32 were also positive for EA-IgA. However, none of the 117 participants with an initial VCA-Ig-negative status were positive for EA-IgA. Compared with VCA-IgA, EA-IgA had a higher seroconversion probability from positive to negative (*P* = 0.001) and a lower probability from negative to positive (*P* < 0.001) (Fig. 3). The median duration of an EA-IgA-positive status was 14 months (95% CI 11.3–16.6 months), and all 32 participants converted to negative in the 5-year follow-up. Of the 1024 participants with an initial EA-IgA-negative status, the 5-year cumulative probability of seroconversion was 19.6% (95% CI 14.3%–24.9%). Furthermore, the cumulative probability of seroconversion for serum EBV antibodies did not differ significantly between males and females (Fig. 3c–f). In the participants with an initial VCA-IgA-positive status, the cumulative probability of seroconversion was 88.1% in males (95% CI 83.2%–93.0%) and 83.0% in females (95% CI 77.7%–88.3%). In the participants with an initial VCA-IgA-negative status, the cumulative probability was 100.0% in males and 82.7% (95% CI 71.9%–93.5%) in females. In the participants with an initial EA-IgA-positive status, the cumulative probabilities were 100.0% for both males and females. In the participants with an initial EA-IgA-negative status, the cumulative probability was 17.7% (95% CI 11.8%–23.6%) in males and 21.0% (95% CI 13.2%–28.8%) in females.

## Discussion

The current findings indicated that the baseline EBV VCA-IgA-positive rate was 7.6% in males and 6.6% in females, and the positive rate tended to increase with age from 6.0% in the 30–39 year age group to 8.4% in the 50–59 year age group. These findings are consistent with the epidemiological characteristics of areas with a high NPC risk, with increased incidence in males and peak incidence among the population aged 50–59 years [3]. Therefore, an age- and sex-dependent high-risk cutoff value for VCA-IgA should be considered. The currently recommended referral criteria for nasopharyngeal endoscopy and biopsy (VCA-IgA ≥1:40 and/or VCA-IgA and EA-IgA double positive) may be appropriate for men of 30–39 years old; however, these criteria become less specific with age and may thus increase the possibility of unnecessary diagnostic tests in participants of 50–59 years old. Larger randomized clinical trials should be conducted to further address this concern.

Titer fluctuations in the EBV antibody serostatus were common in this non-NPC population. In a previous NPC screening study in an endemic area, many non-NPC participants were advised to undergo expensive and invasive nasopharyngeal endoscopy and biopsy examinations as a result of increased serum EBV antibodies [14]. Our findings demonstrated that 55.5% of the participants with an initial VCA-IgA-positive status met the high-risk criteria, whereas 20.6% of the participants with an initial VCA-IgA-negative status met the high-risk criteria at least once during the first 5-year study period. One potential strategy for reducing unnecessary follow-up examinations is to repeatedly test for serum EBV antibodies several weeks before referral for endoscopy and nasopharyngeal biopsy to not only preclude random measurement errors but also eliminate participants who exhibit a short-term EBV antibody increase caused by environmental factors. If the repeated tests indicate that the antibody levels remain increased, a fiberscopic examination or biopsy is recommended. Otherwise, these expensive and invasive examinations may be avoided.

Approximately 85.2% of the participants with an initial VCA-IgA-positive status converted to negative within 5 years. However, we determined that the seroconversion rate of NPC participants from positive to negative was substantially lower (15.4%) in our original study (*P* < 0.001) [17] than the seroconversion rate of non-NPC participants. This finding suggested that serum EBV antibody titer fluctuation may be used to determine the optimal screening interval. Similar to several other NPC screening studies in South China [14, 15], the preset screening interval was 1 year for participants with both VCA-IgA and EA-IgA positive, and the positive predictive value was less than 1% [13, 15]. We speculated that the use of results from only one test to determine the clinical screening interval does not take into account transient increases induced by environmental stressors, which may thus result in many false-positive evaluations. Thus, a rational screening interval for participants with EBV seropositive at baseline may be adjusted by retest results. If the repeated EBV antibody tests remain positive, then a retest in 1 year should be recommended. In contrast, the screening interval may be prolonged in negative cases.

These frequent seroconversions of EBV antibodies may arise from environmental inducers that change throughout an individual’s lifetime. The virus is typically restricted to resting memory B cells by the host immune system; however, it may be periodically reactivated. Following EBV reactivation, it switches from a latent phase to a lytic phase with concomitant expression of viral antigens. The host immune system produces a series of antibodies against viral antigens, especially IgA antibodies against lytic phase antigens, including VCA-IgA and EA-IgA. The living environment may frequently change. When these inducers are eliminated, the virus may return to the latent state in memory B cells, and the serum antibody levels gradually decrease, which results in seroconversion to a negative status.

We determined that the seroconversion rate of EA-IgA from positive to negative was significantly higher than that of VCA-IgA in the first 5-year period (100.0% vs. 85.2%, *P* = 0.001). In contrast, the seroconversion rate of EA-IgA from negative to positive was lower than that of VCA-IgA (19.6% vs. 100.0%, *P* < 0.001). A high-specificity screening marker must yield a low false-positive rate throughout the screening course for the non-disease population. Our previous study [13] and other studies [16, 21] have reported that the baseline EA-IgA-positive status is lower than the VCA-IgA-positive status in the non-NPC population. Furthermore, VCA-IgA exhibited a higher two-way seroconversion rate (from negative to positive and from positive to negative) in the non-NPC population, whereas the EA-IgA conversion was only one-way (from positive to negative). This pattern results in a higher false-positive rate for VCA-IgA and a higher specificity for EA-IgA during follow-up. Thus, EA-IgA may be more specific for NPC screening than VCA-IgA.

This study had several limitations. First, the study was conducted in Sihui County, China, which has a high NPC incidence worldwide. Therefore, the results may not be translated to other populations in medium- or low-incidence areas. However, NPC screening is more likely to succeed if conducted in a high-risk population; thus, these findings are useful for developing screening strategies for this population. Second, the participants recruited for NPC screening may not represent the general population. For example, screening participants may be healthier or more health-conscious than the general population. However, no report to date has demonstrated that the serum EBV antibody status in the non-NPC population is affected by compliance for NPC screening.

In summary, serum EBV antibody levels in a non-NPC population from an endemic area were increased in males and older participants. Thus, an age- and sex-specific cutoff for the anti-EBV antibody level should be considered for NPC screening. Repeated testing indicated that EBV seroconversions were common in the non-NPC population. This finding may contribute to the design of more efficient NPC screening strategies with a rational adjustment of rest-retest intervals.
